# Resting-state functional connectivity in women with PMDD

**DOI:** 10.1038/s41398-019-0670-8

**Published:** 2019-12-11

**Authors:** Nicole Petersen, Dara G. Ghahremani, Andrea J. Rapkin, Steven M. Berman, Noor Wijker, Letty Liang, Edythe D. London

**Affiliations:** 1 Department of Psychiatry and Biobehavioral Sciences, University of California, Los Angeles, Los Angeles, CA 90024 USA; 2 Department of Obstetrics and Gynecology, David Geffen School of Medicine, University of California, Los Angeles, Los Angeles, CA 90024 USA; 3 Department of Molecular and Medical Pharmacology, University of California, Los Angeles, Los Angeles, CA 90024 USA; 4 Brain Research Institute, David Geffen School of Medicine, University of California Los Angeles, Los Angeles, CA 90024 USA

**Keywords:** Psychiatric disorders, Neuroscience

## Abstract

**Background:**

Premenstrual dysphoric disorder (PMDD) is an understudied, debilitating disorder of women. Given evidence for prefrontal cortical and limbic dysfunction in PMDD, we compared intrinsic connectivity of the executive control network (ECN), default mode network (DMN), and amygdala in women with PMDD vs. controls.

**Methods:**

Thirty-six women (18 PMDD, 18 control) participated in fMRI during the follicular and luteal phases of the menstrual cycle. At each time, resting-state functional connectivity was evaluated both before and after participants performed an emotion regulation task. The ECN was identified using independent components analysis, and connectivity of left and right amygdala seeds was also evaluated.

**Results:**

Nonparametric permutation testing identified a cluster in the left middle temporal gyrus (MTG) with significantly stronger connectivity to the left ECN in women with PMDD vs. controls in all four fMRI sessions. Women with PMDD exhibited no difference in functional connectivity between menstrual cycle phases. Amygdala connectivity did not differ between the groups but differed significantly with menstrual phase, with left amygdala connectivity to cingulate cortex being significantly stronger during the follicular vs. luteal phase. Right amygdala connectivity to the middle frontal gyrus was also stronger during the follicular vs. luteal phase, with no group differences. These findings suggest that women with PMDD have different intrinsic network dynamics in the left executive control network compared to healthy controls.

## Introduction

Premenstrual dysphoric disorder (PMDD) is a severe variant of premenstrual syndrome, characterized by debilitating behavioral symptoms, including dysphoric mood, which occur during the luteal phase of the menstrual cycle and abate following the onset of menstruation^1–4^, causing considerable impairments in quality of life^5,6^. Brain imaging studies of task-related activation during fMRI have identified several regions of abnormal function in women with PMDD. During the symptomatic phase, women with PMDD show greater reactivity of the amygdala to negative stimuli and weaker top-down control of this activation compared to healthy controls^7^. Consistent with this observation, lower pre/postcentral gyrus activation and lower dorsolateral prefrontal cortical (dlPFC) activity have been observed when women with PMDD perform an emotion regulation task during the symptomatic phase of the menstrual cycle compared to the asymptomatic phase^8^. The latter finding generally supports previous evidence linking dlPFC activation to the etiology of PMDD^9^, although that evidence was obtained using an ovarian suppression plus add-back hormone protocol, in which women with PMDD had greater dlPFC activation compared to healthy controls while performing a working memory task. Inasmuch as the dlPFC is strongly linked to emotion regulation (for meta-analyses, see refs. ^10^ and ^11^) dysfunction in this brain region could plausibly lead to problems regulating emotions such as those described by women with PMDD^12^.

Resting-state functional connectivity increasingly has been measured to improve understanding of neuropsychiatric disorders^13^. Patients with Major Depressive Disorder (MDD) exhibit stronger connectivity between the subgenual cingulate cortex and the default mode network (DMN)^14^, and between the insula and amygdala as compared with healthy controls^15^. A meta-analysis extended these reports, documenting stronger DMN intra-network connectivity and weaker frontoparietal intra-network connectivity in MDD patients than in healthy control subjects ^16^.

PMDD shares a number of characteristics with MDD, including overlapping diagnostic criteria and comparable impairments in quality of life^17^. The neural features that have been most strongly linked to MDD and other affective disorders have not yet been observed in women with PMDD, and vice versa – but lack of evidence that these neural features overlap cannot be taken as proof that they do not. Some preliminary forays have investigated the neural features of menstrual-related mood disorders, and differences in functional connectivity between women with Premenstrual Syndrome (PMS, a milder syndrome than PMDD) and healthy controls have been observed. Women with PMS have stronger amygdala-prefrontal cortical connectivity, and connectivity between the right amygdala and right precentral gyrus, left ACC, and left medial prefrontal cortex correlates positively with the strength of symptoms in these patients^18^. A network-level analysis using independent component analysis (ICA) to identify the DMN found stronger connectivity between the DMN and both the superior temporal gyrus and precentral gyrus in women with PMS as compared to healthy controls^19^. These reports suggest that network connectivity may differ in women with PMDD compared with healthy controls, and that these differences may point to potential therapeutic targets.

Yet resting-state functional connectivity studies in women with PMDD per se (rather than PMS) had not been performed, and studies in women with milder forms of premenstrual disorders may not be informative regarding PMDD. We therefore compared resting-state functional connectivity in women with PMDD and healthy controls during the premenstrual and follicular phases of the menstrual cycle. Resting-state fMRI scans were performed once before and again after participants completed an emotion regulation task (see Petersen et al.^8^ for details of task methodology and findings). Given previous evidence linking abnormal task-related activation in prefrontal cortical regions to PMDD^8,9^, we selected the executive control network (ECN) as a network of interest. Amygdala and DMN connectivity were assessed due to evidence that menstrual phase^20^, ovarian hormones^21^, and PMDD^22^ may all influence amygdala function, and evidence that DMN connectivity is abnormal in major depression, which shares many symptoms with PMDD ^23,24^.

## Methods and materials

Data from these participants have been previously presented, and additional information regarding materials and methods are available in those publications^8,12^. The study protocols were approved by the UCLA Institutional Review Board, and all participants gave written, informed consent before any study procedures were carried out.

Participants were recruited from the greater Los Angeles community through flyers and Internet advertisements. Eighteen healthy controls and 18 women with PMDD completed the study. Complete data were not available for one participant in the PMDD group, who asked to leave the scanner before completing the session due to discomfort; therefore, data from 17 PMDD participants are included in the imaging results presented below. All participants completed two experimental sessions: one during the follicular phase, 5–12 days after the onset of menstruation, and one during the luteal phase, 10–14 days after ovulation. Ovulation was estimated using at-home urinary luteinizing hormone detection kits (Clearblue® Digital Ovulation kit; SPD Swiss Precision Diagnostics GmbH, Geneva). The order of the first testing session (follicular or luteal) was determined randomly, leading to 61% of PMDD participants and 50% of controls beginning the study in the follicular phase, and the rest in the luteal phase.

### Inclusion and exclusion criteria

The participants were required to be between the ages of 18 and 44 inclusive, non-smokers, fluent in English, right-handed, willing to use non-hormonal contraception or abstinence for the duration of the study, and to have regular menstrual cycles every 24–32 days. Participants were excluded if they reported any history of psychiatric diagnoses other than unipolar depression, or if unipolar depression had occurred within the past two years. Mental health history was assessed by a trained clinician using the Structured Clinical Interview for DSM-IV Axis I Disorders, Patient Edition^25^. Participants were also excluded if they endorsed a history of central nervous system, cardiovascular, hepatic, renal, endocrine, or autoimmune disease during the health history taken by a nurse practitioner. MRI contraindications, including non-removable metal or greater than minimal claustrophobia, were also exclusionary.

A PMDD diagnosis (for inclusion in the PMDD group) was assigned on the basis of scores on the Daily Record of Severity of Problems (DRSP) over the course of two complete menstrual cycles. Diagnosis and subsequent inclusion decisions were made before participants were scheduled for additional data collection. Complete DRSPs were filled out daily online throughout the duration of the study ^26^; graphical depictions of overall symptom profiles for each participant are available as Supplementary Figs. S1–S18. Participants were required to endorse low (<3) scores on all DRSP items during the follicular phase, defined here as 7–12 days after the onset of menstruation, and high scores on DRSP items during the premenstrual phase, defined here as 6 days before menstruation. High scores were operationalized as:

1. scores ≥3 for at least 4 days, and ≥4 for at least 2 days on mood symptoms (DRSP items 1 through 4), and
2. scores ≥3 for at least 2 days, and ≥4 for at least 2 days on at least 5 of symptoms (DRSP items 1 through 11)
3. scores ≥3 for at least 2 days, and ≥4 for at least 2 days on items measuring severity of impairment (DRSP items 12 through 14)

Participants in the healthy control group were required to endorse symptom scores <3 during the premenstrual phase, and <3 during the follicular phase. Absolute values on each day were evaluated rather than averaging together scores across each phase or cycle.

At each testing session, blood samples (5 mL) were collected by venipuncture for assay of progesterone levels by electrochemiluminescence (Roche Elecsys Immunoassay system, F. Hoffman-La Roche, Basel, Switzerland).

Detailed demographic information is presented in Supplementary Table S1. The ethnic composition of each group was somewhat different, with the PMDD group predominantly (78%) non-Hispanic white, and no group predominating among controls (Supplementary Table 1). Statistical comparisons were not performed between groups because of the small cell sizes involved. Group differences in self-reported years of education, self-reported annual income, and IQ estimated by performance on the Shipley-2 Vocabulary Test were tested in a two-way *t*-test in JMP(R) Pro 11.0.0 (SAS Institute Inc., Cary, NC, USA).

### Study design specifications

The sample size (*N* = 18/group) was determined prospectively, and was similar in size to existing published studies evaluating brain activity in women with PMDD^9,22^. Participants completed two fMRI resting-state scanning sessions on each test day separated by an emotion-regulation task. Details of the task and results have been reported (Petersen et al., 2017). Briefly, the task involved using a cognitive distancing strategy to reappraise negative stimuli. Participants were instructed that if they saw the instruction “Far”, they were to imagine that they were far away from the scene they were about to view, as if they were distant observers or reporters reporting on the details and facts of the scene. If they saw the instruction “Close,” they were to imagine that they were actually immersed in the scene they were about to view. Following the instruction, they were presented with either a negatively or neutrally valenced image, selected mostly from the International Affective Picture System. After each such trial (instruction + image), participants rated how bad they felt on a scale from 1 to 4, with 1 indicating “not at all bad” and 4 indicating “very bad” using a hand-held button box in the scanner.

The resting-state scans (152 T2*-weighted echoplanar images; repetition time = 2 s; echo time = 30 ms; slice thickness = 4 mm; flip angle = 90°; matrix: 64 × 64; field of view = 192 mm) were acquired over 5 min with a 3-T Siemens AG Trio MRI scanner (Erlangen, Germany). Participants viewed a black screen and were instructed to, “relax, try to stay as still as possible, and keep your eyes open.” A T1-weighted magnetization-prepared rapid-acquisition gradient echo scan (MPRAGE) and a T2-weighted matched-bandwidth anatomical scan were acquired to improve registration to standard space.

### Preprocessing and analysis

All MRI preprocessing and analyses were performed in the FMRIB Software Library (FSL) version 5.0.9 (www.fmrib.ox.ax.uk/fsl). Non-brain matter was removed with FSL’s brain extraction tool, low-frequency trends were removed with high-pass filtering (100 s threshold), and functional images were registered to standard space through three steps^1^: FMRIB's Linear Image Registration Tool (FLIRT) was used to register the resting-state functional scan to the matched-bandwidth image through affine transformation^2^; the same procedure was used to register the resting-state functional image to the MPRAGE image; and finally^3^ this image was transformed to the MNI152 template through FMRIB's Nonlinear Image Registration Tool (FNIRT) with 12 degrees of freedom and a warp resolution of 10 mm.

Motion cleaning was performed with regressors entered as confound variables into a linear regression model. First, twenty-four motion regressors were used as per^27^. These included the three translational motion parameters along the X, Y, and Z axes and three in the rotational dimension (“pitch”, “roll” and “yaw”) (6 total); then the temporal difference (difference of the current and previous time-point) (6 total), and the quadratics of these 12. Next, framewise displacement (FD) was entered as “spike” regressors in the model^28,29^. To account for additional variance due to noise^30^, the global signal was included in the regression model^31^. The residuals produced by the regression model were then scaled and normalized at each voxel with the equation: [(residuals–mean)/standard deviation] + 100.

To examine intrinsic ECN connectivity, preprocessed images were entered as multi-session temporally concatenated data into FSL’s Multivariate Exploratory Linear Optimized Decomposition into Independent Components (MELODIC) tool with dimensionality estimation set to 20 components. The components of interest for further analysis (left and right ECN) were identified through visual inspection (similarity to intrinsic connectivity networks 15 and 18 in Laird et al.^32^) Individual subject-level maps of these components were determined using FSL’s dual regression tool. Effects of group (PMDD vs. control) and phase (follicular vs. luteal) on ECN connectivity were tested using Randomise, FSL’s nonparametric permutation-based statistical modeling tool^33^, using Threshold-Free Cluster Enhancement (TFCE), a cluster-extent threshold of *Z* = 2.3 and 10,000 permutations in a mixed-model design with group entered as a between-subjects factor and phase as a within-subjects factor. The group-by-phase interaction was evaluated by testing for an effect of group on difference maps of the follicular-luteal differences. Group and phase effects on resting-state functional connectivity were tested on the scan that preceded the task (rest 1), and then the same analysis was repeated on the scan that followed the task (rest 2). Using the same model, exploratory analyses were also performed on a DMN component (see https://neurovault.org/collections/4829/ for spatial map of this component).

Seed-based connectivity of the left and right amygdala was derived using the General Linear Model as implemented in FSL’s FEAT. First, amygdala regions of interest defined by the Harvard-Oxford Probabilistic Atlas were transformed into each participant’s native space. Time series data within the left and right amygdala were extracted from the motion-cleaned data (described above) and averaged across voxels, resulting in a single-time series variable for each of the two (left and right) amygdala regions. These time-series were separately entered into two GLM models, one for the right and one for the left amygdala, resulting in a “connectivity map” (GLM parameter estimate maps) for each of the two amygdala regions for each participant. These maps were analyzed for group and phase effects using Randomise with TFCE, 10,000 permutations, and a cluster-extent threshold of *Z* = 2.3. The main effects of group and phase and their interaction were evaluated in the same manner as the ECN data (described above).

To test whether differences in connectivity were related to PMDD symptoms (i.e., average of total DRSP score during the symptomatic phase), the averaged parameter estimates from the dual-regression model were extracted from clusters where group differences in resting-state functional connectivity were detected. These values were entered into a linear mixed model as a fixed effect, and participant as a random effect. Because PMDD symptoms were nearly zero in controls, the analysis was performed only on data from women with PMDD. PMDD symptoms as reported on the DRSP were entered as the dependent variable in the model.

A similar model was used to test whether functional connectivity was related to successful emotion regulation. Connectivity strength between the left ECN and the cluster that differed between groups was extracted and entered into a linear mixed model as a fixed effect, with participant as a random effect. Here, group (PMDD vs. control) was entered as a covariate. To evaluate the effect of connectivity strength on emotion regulation, mean participant ratings for all trials in which they viewed negatively valenced stimuli and were instructed to using a distancing strategy (“negative, far” trials) were entered into the model as the dependent variable.

## Results

### Demographics

The groups did not differ significantly in age, years of education, income, or IQ as estimated by the Shipley-2 vocabulary test^34^, all *p*s > 0.1 (see Supplementary Table S1).

### Progesterone

Progesterone levels differed significantly from the follicular to luteal testing days [*F*(1,71) = 44.52, *p* < 0.0001] but not between the PMDD and control groups during either the follicular [*F*(1,35) = 2.25, *p* = 0.14] or luteal [*F*(1,35) = 0.38, *p* = 0.54] phase.

### Network identification

The ECN was visually identified from the 20 components generated by MELODIC. A left-lateralized frontoparietal network was identified as the left ECN, and a right-lateralized frontoparietal network was identified as the right ECN (Supplementary Fig. S19; all images presented in radiological orientation).

### Group differences in resting-state functional connectivity

Effects of group and phase were tested in a mixed-model design for the left ECN, right ECN, and default mode network. In both the phase comparison (follicular vs. luteal) and the group-by-phase interaction test, no voxels survived permutation testing at the 0.01 level, suggesting that there was no effect of phase and no group-by-phase interaction on resting-state functional connectivity within the left ECN. We employed an additional layer of correction for the 5 comparisons performed (left ECN, right ECN, DMN, right amygdala, left amygdala), further bringing the significance threshold for these familywise-error corrected clusters down to *p* = 0.01 (*α* = 0.05/5). No effects of group, phase, or their interaction were found in the right ECN or DMN.

**Fig. 1:**
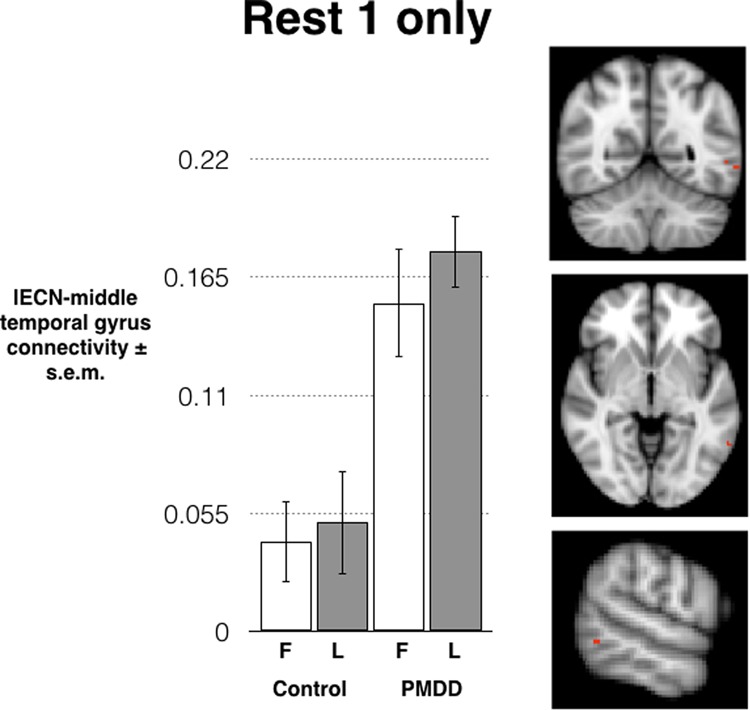
**Connectivity between the ECN and a small cluster (4 non-contiguous voxels) in the middle temporal gyrus was significantly stronger in the PMDD group compared to healthy controls.** This was equally true in both the follicular and luteal phase.

In the left ECN analysis only, a significant main effect of group emerged in a very small functional group of four non-contiguous voxels in the left middle temporal gyrus (peaks of two-voxel clusters at MNI *x*, *y*, *z* = −60, −54, −4 and −54, −56, 0), indicating significantly stronger connectivity between these middle temporal gyrus voxels and the rest of the left ECN in the PMDD group compared to healthy controls, irrespective of menstrual phase. The GLM parameter estimates in these voxels were extracted and the means for each group and phase were plotted (Fig. 1). No effects of group or phase were found in right ECN. Notably, this cluster was not significant after correcting for the number of tests performed (*p* = 0.036).

**Fig. 2:**
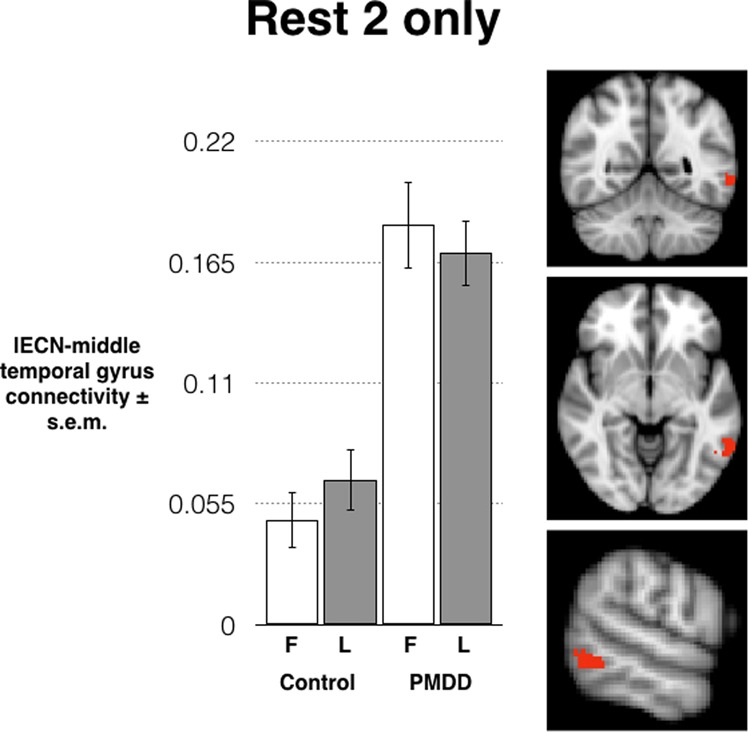
**Following the emotion regulation task, a second resting-state scan was performed.** Here, a larger cluster overlapping the one observed in the first resting-state scan again showed stronger connectivity with the ECN in the PMDD group compared to controls, irrespective of menstrual phase.

When the same analysis was repeated for the second rest scan (following the emotion regulation task), the same relationship was observed: significantly stronger connectivity between a middle temporal gyrus cluster and the rest of the left ECN in the PMDD group compared to controls, irrespective of menstrual phase. The cluster showing a significant group difference included 112 voxels (peak voxel at −58, −60, −10). Again, there was no effect of phase or group-by-phase interaction on resting-state functional connectivity in the left or right ECN (Fig. 2). This cluster survived correction for the total number of tests performed (*p* = 0.002).

**Fig. 3:**
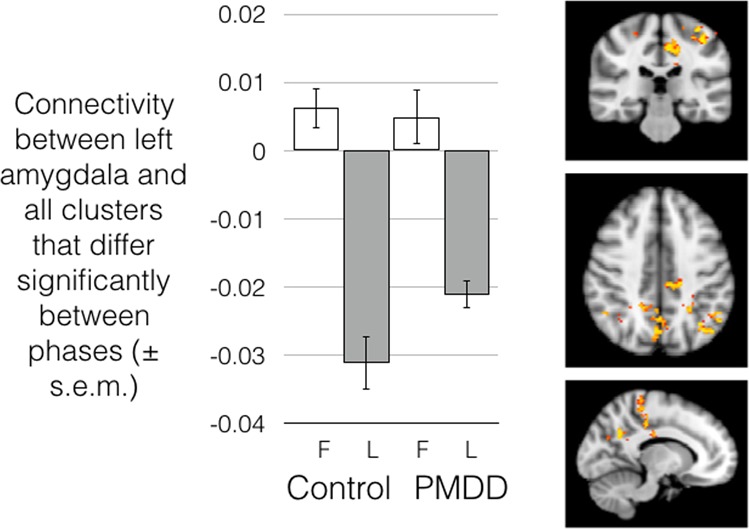
Left amygdala connectivity with a number of clusters throughout the posterior and mid-cingulate, and parietal cortex, was significantly higher in the follicular compared to luteal phase in both controls and women with PMDD.

A seed-based connectivity analysis comparing connectivity of the amygdala in the follicular vs. luteal phases showed significantly stronger connectivity between the left amygdala and a number of parietal and midline clusters focused around the posterior cingulate cortex, mid-cingulate cortex, and right angular gyrus in the follicular phase. A mixed-model analysis revealed a significant group-by-phase interaction, with a smaller follicular-luteal change in connectivity in women with PMDD compared to healthy controls (Fig. 3; Supplementary Table S2). This follicular-luteal connectivity difference was only observed in the first fMRI session of the day, before the emotion regulation task was performed. No effects of group or phase were found during the second, post-task resting-state session.

**Fig. 4:**
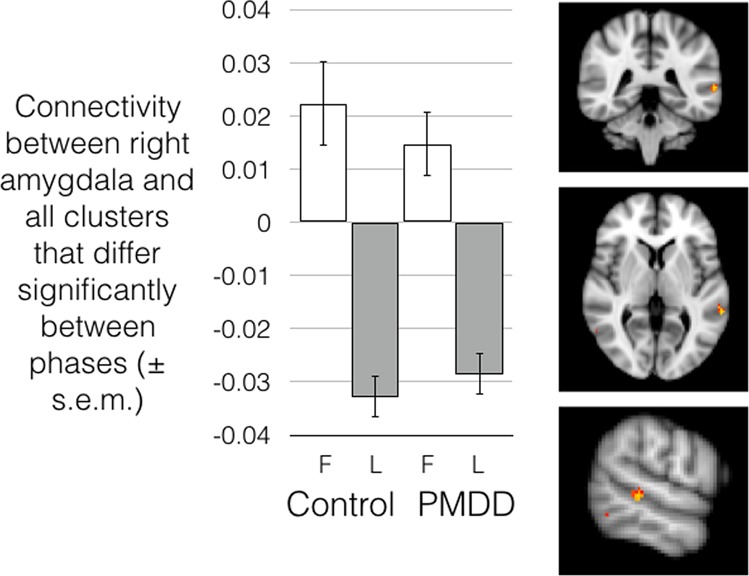
Right amygdala connectivity with clusters in the cerebellum and middle temporal gyrus, was significantly stronger in the follicular phase compared to the luteal phase in controls as well as women with PMDD.

Similarly, a seed-based connectivity analysis evaluating right amygdala connectivity also indicated stronger connectivity between the right amygdala and clusters in the middle temporal gyrus in the follicular phase than the luteal phase that survived correction for the total number of comparisons (Fig. 4; Supplementary Table S3). No effect of group or group-by-phase interaction was detected, and this menstrual phase effect was observed only in the first resting-state session, which took place before the emotion regulation task.

### Linking resting-state connectivity to behavior

To test whether connectivity of the MTG cluster to lECN was related to PMDD symptom severity, the mean parameter estimates of the cluster in both rest 1 and rest 2 were extracted and correlated with PMDD symptoms as reported on the DRSP. No significant relationship was observed (*p* = 0.68).

**Fig. 5:**
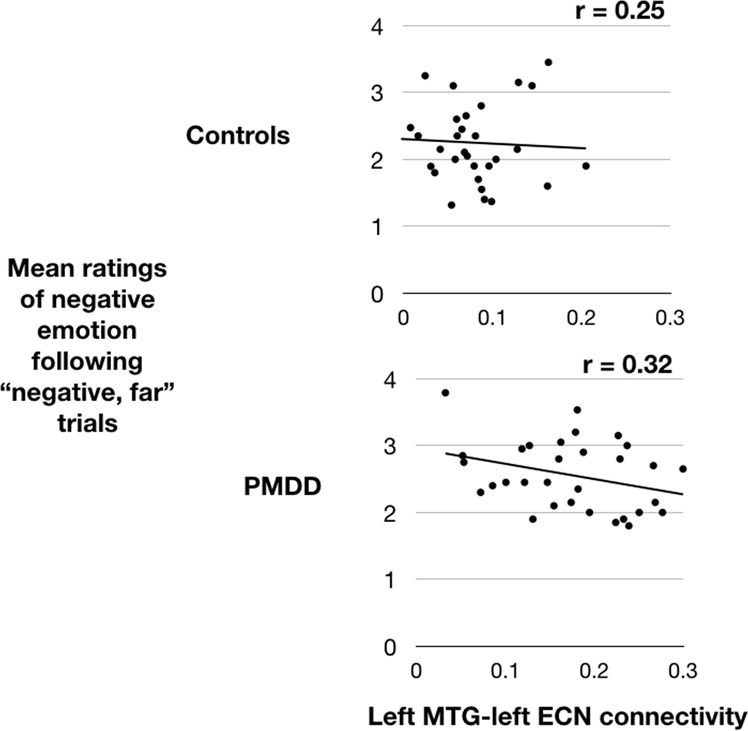
**Connectivity between the left ECN and a cluster in the left middle temporal gyrus was significantly stronger in women with PMDD compared to controls, and ostensibly related to emotion regulation in the PMDD group.** After the emotion regulation task, connectivity between the left ECN and this cluster correlated negatively with “negative, far” ratings during the task, implying that stronger connectivity reduced negative emotion produced by the task.

The relationship between this connectivity measurement and successful emotion regulation through cognitive reappraisal was also tested using this model. The mean parameter estimates of this cluster during rest 1 were unrelated to ratings on “negative, far” trials (*p* = 0.18) but were significantly related during rest 2 (*p* *=* 0.0054). This relationship (Fig. 5) was significant in the PMDD group (*p* = 0.04) but not the control group (*p* = 0.28).

Left and right amygdala connectivity was not related to behavior on the emotion regulation task.

### Counterbalancing

To verify that order effects did not influence the results, a linear mixed model testing the main effect of session (timepoint 1 versus timepoint 2) was run; no effects of session on any resting-state functional connectivity parameter were observed.

### Effect size maps

Effect size maps illustrating the outcome of all analyses described above are available at https://neurovault.org/collections/4829/.

## Discussion

Researchers have speculated that PMDD symptoms may be explained by “impaired ovarian hormones-mediated sensitivity of emotional and cognitive brain networks”^35^, yet the integrity and dynamics of such networks in women with PMDD have been only minimally explored. Neural models of affective disorders have started to converge on findings that network-level dynamics may underlie affective symptoms, with top-down control from prefrontal cortical regions failing to effectively regulate bottom-up affective processes from limbic regions (for review, see Disner et al.^36^) to produce these symptoms. Here, we show that intrinsic network connectivity in women with PMDD is broadly consistent with this model of prefrontal cortical dysregulation insofar as women who meet DSM-5 criteria for PMDD have significantly stronger connectivity between a region of the left middle temporal gyrus and the left ECN relative to healthy controls when challenged by an emotional task. This effect appears to be stable across menstrual cycle phases.

The cluster that shows a group difference in connectivity appears to be related to regulation of negative emotion in women with PMDD only, in whom the mean parameter estimates of this cluster correlated with ratings of negative emotion obtained during an emotion regulation task. The negative direction of the correlation implies that stronger connectivity between this cluster and the rest of the left ECN enables more successful emotion regulation. Although it may be tempting to speculate that greater connectivity in women with PMDD should produce better emotion regulation compared to controls, the effect observed here points to a potential compensatory or alternate pathway to engage cortical resources for emotion regulation in women with PMDD for several reasons^1^: it was observed only in women with PMDD (not controls)^2^, the cluster that differed between women with PMDD and controls increased in size after participants performed an emotionally demanding task, and^3^ the connectivity of this cluster differed considerably between women with PMDD and controls.

These findings are also broadly consistent with previous evidence showing differences in intrinsic network connectivity between healthy controls and women with PMS, a milder syndrome similar to PMDD. Similar to our finding of stronger connectivity between the left middle temporal gyrus and left ECN compared to controls, Liu et al.^18^ reported stronger connectivity between the left middle temporal gyrus and default mode network in women with PMS compared to controls. Intriguingly, in both studies, the effect was independent of menstrual cycle phase, despite the dramatic difference in symptom presentation between the follicular and late luteal phases.

The seed-based connectivity analysis did not indicate a significant effect of PMDD on amygdala connectivity. Instead, it revealed a menstrual phase-related shift in amygdala connectivity as the cycle progressed from the follicular to late luteal phases in both healthy controls and women with PMDD. Left amygdala connectivity to the posterior cingulate cortex, mid-cingulate area, right angular gyrus, and right superior parietal cortex was significantly weaker in both groups (controls and PMDD) during the late luteal phase compared to the follicular phase. Similarly, right amygdala connectivity to the cerebellum and left middle temporal gyrus were significantly weaker during the late luteal phase compared to the follicular phase. Because these connectivity reductions were observed in healthy controls as well as women with PMDD, they appear to reflect normal cyclicity in brain connectivity. The functional significance of this change is not evident from these data, and warrants further investigation.

Some limitations affected this study. Resting-state connectivity datasets are information-rich and provide the opportunity to test for effects within many networks, as well as between any number of seed regions. The small sample size available here provided limited statistical power, which constrained the number of hypotheses that could be tested; therefore, we limited hypothesis testing to the highest priority networks and seeds. We have made these data available for other investigators to search for trends or test their own hypotheses (see https://neurovault.org/collections/4829/). It also bears considering that the small sample may obscure effects produced by subtypes of PMDD, as the group was too small to further subdivide. Since these data were collected, temporal subtypes reflecting unique timecourses of symptom presentation (i.e., symptoms constrained to a relatively brief premenstrual window; symptoms persisting through the entire luteal window; or symptoms that persist into the onset of the next cycle) have been described^37^. These and other subtypes of PMDD patients may experience different patterns of intrinsic network connectivity that could not be addressed in this study and represent a target for future investigations.

The data presented here point to a fundamental neurobiological difference between women with PMDD and healthy controls. Identifying the network dynamics that manifest differently in women with PMDD moves the field toward identifying a biomarker for this under-researched condition^6^. As experts in the field have called for novel therapeutic approaches^38^, this report identifies targets for brain-based therapies, such as neurostimulation or neurofeedback, which have shown promise for treatment of other affective disorders.

## Supplementary information

Supplement
