# Supplementary material for: Resting-state functional connectivity in women with PMDD
Source: Transl Psychiatry. 2019 Dec 11;9:339. doi: 10.1038/s41398-019-0670-8 (PMC6906514; doi:10.1038/s41398-019-0670-8)
Supplement: Supplementary file 1 — Supplement [file 41398_2019_670_MOESM1_ESM.docx]

**Resting-state functional connectivity in women with PMDD: Supplemental Tables and Figures**

|  | *Control group* | *PMDD group* | *Control vs. PMDD* |
| --- | --- | --- | --- |
|  |  |  | *p-value* |
|  |  |  |  |
| Age (yr., mean ± SD) | 25.4 ± 7.0 | 29.2 ± 7.2 | 0.12 |
|  |  |  |  |
| Ethnicity |  |  | n/a |
| >1 ethnic group | 3 | 0 |  |
| Asian | 3 | 3 |  |
| Black | 2 | 1 |  |
| Hispanic | 6 | 0 |  |
| White (non-Hispanic) | 4 | 14 |  |
|  |  |  |  |
| Education (yr., mean ± SD) | 15.6 ± 2.7 | 16.7 ± 3.6 | 0.30 |
|  |  |  |  |
| Income (mean ± SD) | 3.71 ± 3.8 | 4.44 ± 3.5 | 0.56 |
|  |  |  |  |
| Shipley-2 vocabulary score | 74.7 ± 12.6 | 79.7 ± 10.2 | 0.21 |
| (% correct, mean ± SD ) |  |  |  |

**Supplemental Table S1:** Demographic information describing the control and PMDD participants and comparisons between the two groups. Ethnicity was described by self-identification. Yearly estimated income was reported in increments of ten thousand dollars (e.g., 0 = $0-$10,000; 1 = $10,001-$20,000; 2=$20,001-$30,000; etc.).

|  |  | *Location of* | | *Brain region* | | *Corrected p-* |
| --- | --- | --- | --- | --- | --- | --- |
| *Number of Voxels* | | *peak voxel* | |  |  | *value* |
|  |  | *(X, Y, Z)* | |  |  |  |
|  |  |  |  |  |  |  |
| 2656 |  | 16, -42, 34 |  | Posterior |  | 0.002 |
|  |  |  |  | cingulate |  |  |
|  |  |  |  | cortex |  |  |
|  |  |  |  |  |  |  |
| 54 |  | -6, -8, 20 |  | Cingulate | | 0.004 |
|  |  |  |  | gyrus | |  |
|  |  |  |  |  |  |  |

**Supplemental Table S2:** Clusters with significantly stronger connectivity of the left amygdala during the follicular compared to luteal phase (only clusters with >15 voxels reported).

| *Number of Voxels* | *Location of* | | *Brain region* | | *Corrected p-value* | |
| --- | --- | --- | --- | --- | --- | --- |
|  | *peak voxel* | |  |  |  |  |
|  | *(X, Y, Z)* | |  |  |  |  |
|  |  |  |  |  |  |  |
| 31 | -62, -40, 2 |  | Left middle temporal |  | 0.008 |  |
|  |  |  | gyrus |  |  |  |
| 16 | -58, -54, |  | Left middle temporal | | 0.01 |  |
|  | -10 |  | gyrus | |  |  |

**Supplemental Table S3:** Clusters with significantly stronger connectivity of the right amygdala during the follicular compared to luteal phase (only clusters with >15 voxels reported).


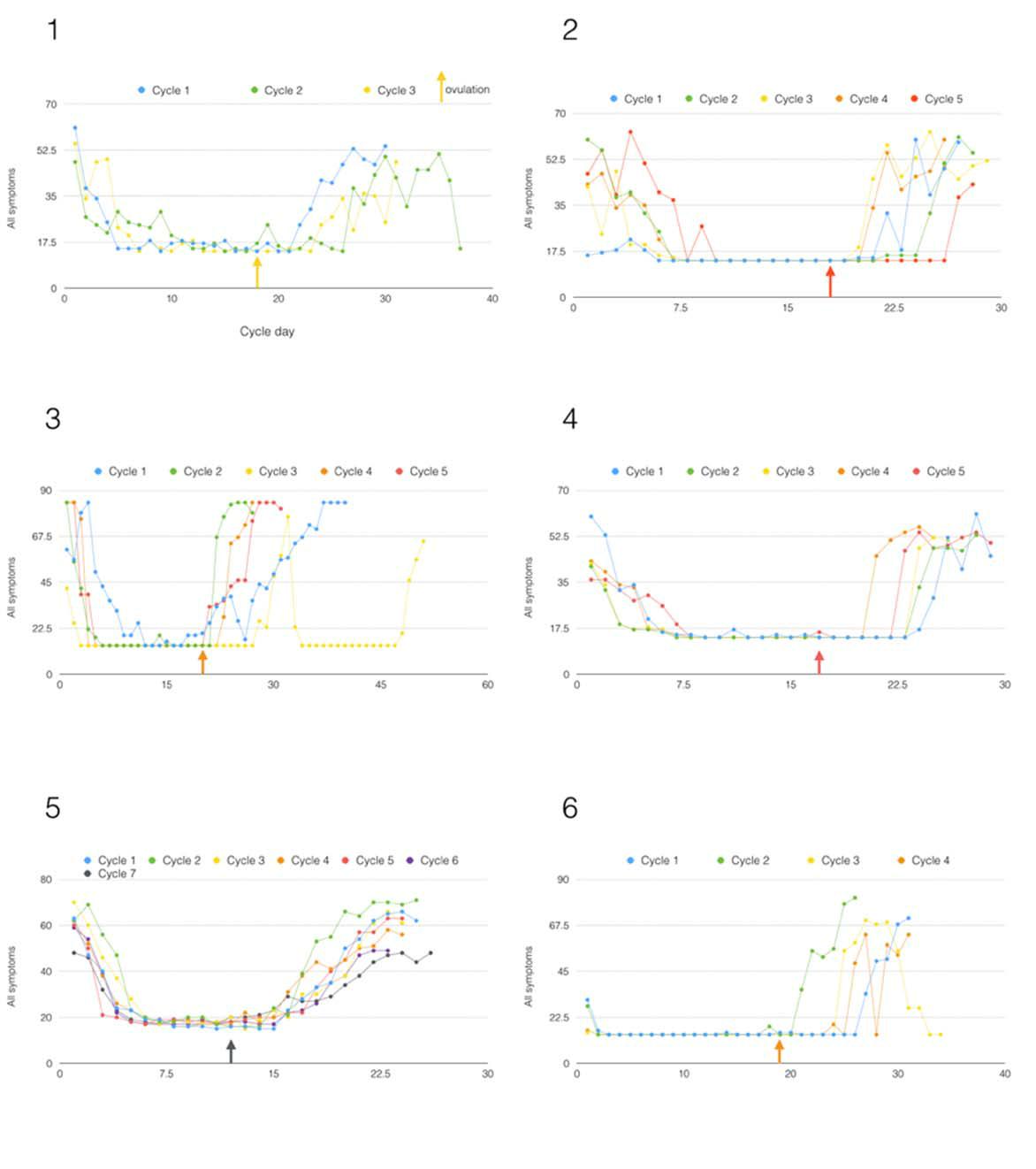


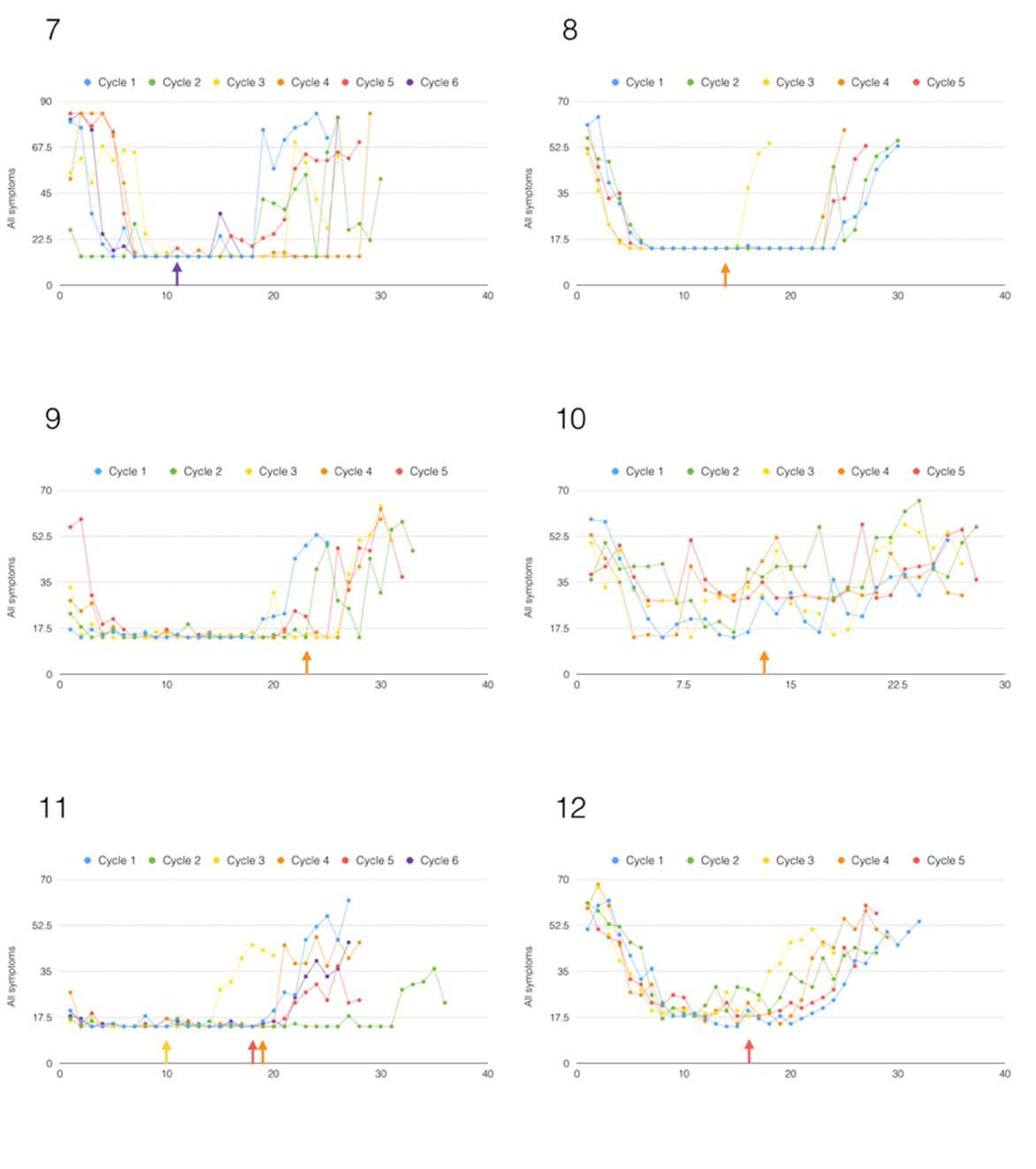


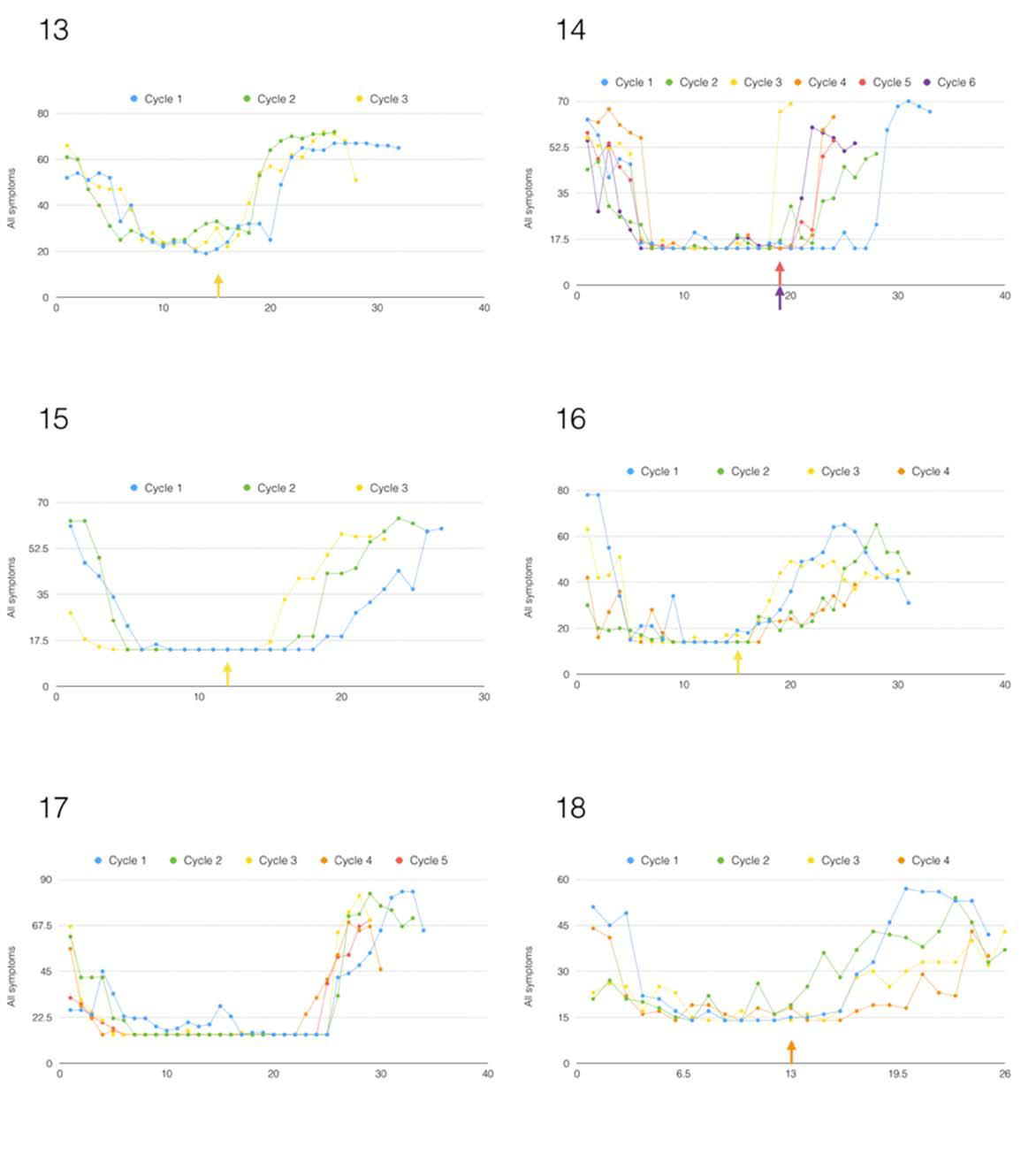


**Supplemental figures S1 through S18:** Symptom profiles describing each of the 18 PMDD participant’s symptoms are shown. These data reflect the summation of all symptoms reported on the DRSP for each day that a participant was in the study. Each colored line reflects a different menstrual cycle for each participant. An arrow indicates the approximate date of ovulation measured with luteinizing hormone urinalysis; the color of this arrow matches the color of the line depicting the cycle with which that ovulation date was associated (e.g., a yellow arrow on cycle day 18 and yellow line depicting cycle 3 indicates that ovulation took place on cycle day

18 during cycle 3). Additional details of daily symptoms are available for data sharing by request.


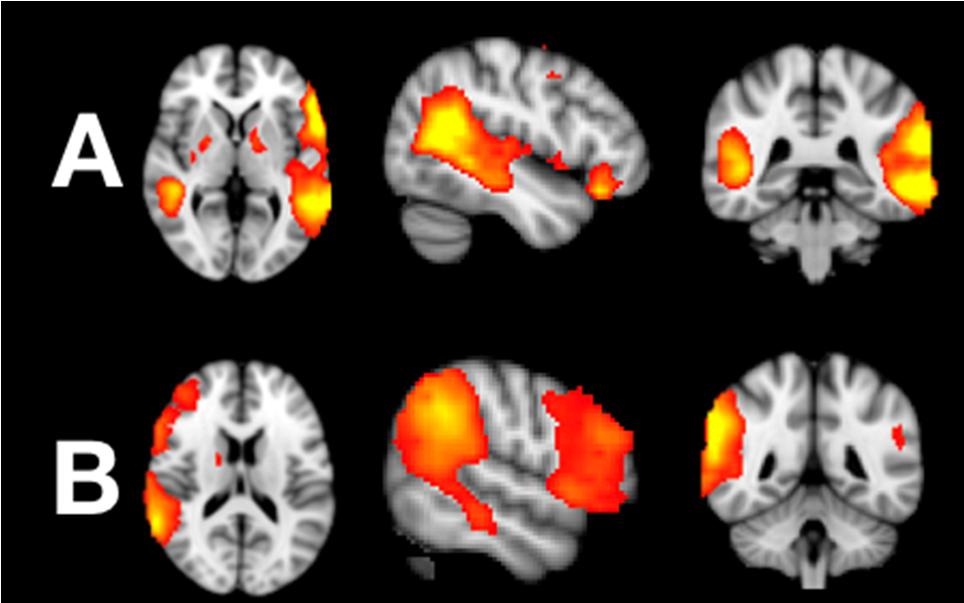


**Supplemental figure S19:** The ECN was identified through ICA and visual inspection. In these data, the ECN was decomposed into left (top) and right (bottom) components.
